# Suitability of Ex Vivo-Expanded Microtic Perichondrocytes for Auricular Reconstruction

**DOI:** 10.3390/cells13020141

**Published:** 2024-01-12

**Authors:** Yvonne Jakob, Johann Kern, David Gvaramia, Philipp Fisch, Ralph Magritz, Sven Reutter, Nicole Rotter

**Affiliations:** 1 Department of Otorhinolaryngology Head and Neck Surgery, Medical Faculty Mannheim, Heidelberg University, Theodor-Kutzer-Ufer 1-3, D-68167 Mannheim, Germany; johann.kern@medma.uni-heidelberg.de (J.K.); david.gvaramia@medma.uni-heidelberg.de (D.G.); nicole.rotter@umm.de (N.R.); 2 Tissue Engineering and Biofabrication Laboratory, Department of Health Sciences & Technology, ETH Zurich, Otto-Stern-Weg 7, CH-8093 Zurich, Switzerland; philipp.fisch@hest.ethz.ch; 3 Clinic for Otorhinolaryngology, Oberhavel-Kliniken GmbH, Klinik Henningsdorf, Marwitzer Strasse 91, D-16761 Henningsdorf, Germany; ralph.magritz@oberhavel-kliniken.de

**Keywords:** perichondrocytes, microtia, tissue engineering, auricular reconstruction, 3D cell cultures, spheroids

## Abstract

Tissue engineering (TE) techniques offer solutions for tissue regeneration but require large quantities of cells. For microtia patients, TE methods represent a unique opportunity for therapies with low donor-site morbidity and reliance on the surgeon’s individual expertise. Microtia-derived chondrocytes and perichondrocytes are considered a valuable cell source for autologous reconstruction of the pinna. The aim of this study was to investigate the suitability of perichondrocytes from microtia patients for autologous reconstruction in comparison to healthy perichondrocytes and microtia chondrocytes. Perichondrocytes were isolated via two different methods: explant culture and enzymatic digestion. The isolated cells were analyzed in vitro for their chondrogenic cell properties. We examined migration activity, colony-forming ability, expression of mesenchymal stem cell markers, and gene expression profile. We found that microtic perichondrocytes exhibit similar chondrogenic properties compared to chondrocytes in vitro. We investigated the behavior in three-dimensional cell cultures (spheroids and scaffold-based 3D cell cultures) and assessed the expression of cartilage-specific proteins via immunohistochemistry, e.g., collagen II, which was detected in all samples. Our results show that perichondrocytes from microtia patients are comparable to healthy perichondrocytes and chondrocytes in terms of chondrogenic cell properties and could therefore be a promising cell source for auricular reconstruction.

## 1. Introduction

Microtia, a congenital anomaly of the external ear, covers a spectrum of phenotypes ranging from mild deformity with a smaller pinna to the complete absence of the external ear (anotia) [1,2,3,4]. The deformity can cause psychological distress due to actual or perceived disfigurement and has an impact on psychosocial functioning, but it can also lead to physical impairment, including hearing loss [1,3,5]. The current gold standard of microtia treatment is autologous reconstruction, in which cartilage grafts from the rib are used to form an auricle [6,7,8,9]. However, the procedure is extremely challenging, and the outcome may vary depending on various factors such as the skill of the surgeon or the amount of available rib cartilage [4,10,11]. In addition, calcification of the rib cartilage may occur, which affects the carved auricular frame and makes the reconstructed ear stiffer [12,13]. Furthermore, donor-site morbidities such as visible chest deformity or even pneumothorax, as well as post-operative infections at both operative sites, can occur [4,14]. The use of alloplastic materials composed of silicone or porous polyethylene overcomes the problem of donor-site morbidity but harbors risks for other complications including inflammation, implant fracture, erosion, or extrusion [4,14,15].

Tissue engineering (TE) technologies offer new opportunities for the treatment of microtia as they enable the production of cartilage tissue implants in vitro using autologous cells and biomaterials [16,17,18]. The use of hydrogels for various applications such as tissue engineering, cell-based therapies, or regenerative medicine in general is of great interest [19]. Several groups have demonstrated the feasibility of producing cartilage tissue with shape and mechanical properties comparable to a human auricle [12,20,21,22,23,24,25,26,27]. However, for a large construct like a human auricle, a considerable number of cells, with estimates ranging from 100 to 250 million, are required [12,28]. As it is impossible to obtain such large cell numbers from a small biopsy, an extensive in vitro expansion of the isolated cells is needed before they can be used for tissue engineering. However, chondrocytes dedifferentiate during expansion and progressively lose their chondrogenic phenotype after repeated passaging [29,30,31,32]. Thus, chondrocytes can only be expanded for a certain number of passages before they completely lose their potential to redifferentiate [33,34].

Besides cartilage, an alternative source of chondrogenic cells is the perichondrium, a fibrous tissue that covers most types of non-articular cartilage, including the elastic cartilage of the auricle [35]. Among other functions, the perichondrium is responsible for supplying the cartilage tissue with oxygen and nutrients [35]. It contains fibroblast-like cells called perichondrocytes and is thought to be a source of chondrogenic progenitors [36,37,38]. Several studies have demonstrated the chondrogenic capacity of perichondrocytes either in animal [37,38] or human studies [36,39]. However, the use of the perichondrium as a cell source for TE applications is uncommon due to its limited anatomical availability, and the requirement for its preservation during reconstructive surgery to avoid the disruption of vascular supply to the cartilage at the site of surgical intervention.

The remnant of auricular cartilage in microtia has been considered as an autologous cell source for tissue engineering applications. Several groups have characterized chondrocytes and chondrogenic progenitor cells (CPCs) from microtic cartilage for their usability in the fabrication of tissue-engineered grafts for auricular reconstruction [10,40]. However, the results have been contradictory; while some groups reported robust cartilage formation by chondrocytes from microtic tissue [20,40], others demonstrated their inferior chondrogenic capacity and the formation of disorganized structures in 3D cell culture models [41,42].

Unlike the reconstructive procedures in healthy tissues, in microtia patients, perichondrium is removed together with microtic cartilage and could potentially be readily used as a cell source for tissue engineering [36,39,43]. A study by Kobayashi et al. demonstrated that microtic perichondrocytes are more proliferative and clonogenic in comparison to microtic chondrocytes and can form a tissue containing both cartilage and a fibrous perichondrium-like layer, suggestive of progenitor cell content [40]. Microtic perichondrium may thus be an optimal additional or even an alternative source for the production of TE constructs as compared to microtic cartilage. However, unlike microtic chondrocytes and CPCs, which were directly compared to their healthy counterparts [41,42], a systemic comparison of microtic perichondrocytes with cells from healthy auricular perichondrium has not yet been performed.

In this study, which was part of a Sinergia project funded by the Swiss National Science Foundation with the aim of developing a 3D bioprinted auricle construct for the treatment of microtia patients, we compared progenitor-like characteristics, as well as the chondrogenic potential of perichondrocytes from microtia patients and healthy donors. Specifically, migration, colony-forming capacity, and the expression of progenitor surface markers [44] were investigated. To compare the influence of different isolation methods on cell properties, perichondrocytes were obtained from healthy and microtia tissue both via standard enzymatic digestion and via cell outgrowth from explant cultures, which is a common method for CPC isolation from cartilage tissue. Isolated cells (microtic and healthy perichondrocytes) were analyzed for gene expression of cartilage-specific proteins and their chondrogenic potential in two different 3D cell culture settings (spheroids and scaffold/hydrogel-based). For the scaffold-based model a hydrogel, hyaluronan transglutaminase (HATG)-alginate (Alg) hydrogel (HATG-Alg), developed by our project partners of the ETH Zurich, was used. Since high cell numbers are required for TE applications, isolated cells must be expanded over several passages in cell culture. To identify passage-specific phenotypic changes that can decisively influence the usability of the cells for TE applications, we analyzed the effects of cell culture on the phenotype of microtic and healthy perichondrocytes over four passages. In addition, perichondrocytes from microtia patients were also compared regarding their progenitor-like and chondrogenic properties with cells from the microtic cartilage tissue of the same donor (microtic chondrocytes) to avoid donor variations.

## 2. Materials and Methods

### 2.1. Cell Isolation and Expansion

Microtia cartilage samples (*n* = 10, patients aged 7–32) were obtained from ear reconstruction surgeries, and healthy auricular cartilage samples (*n* = 5, aged 7–88) were obtained from excess tissue from reconstructive surgeries (e.g., tympanoplasty). The collecting and processing of all patient material was approved by the ethics committee of the Medical Faculty Mannheim (number: 2018-584-N-MA).

*Chondrocytes (CC-M)*: The perichondrium was removed from the microtic cartilage. Cartilage was diced into small pieces (<1 mm^3^) and digested overnight (16–18 h) at 37 °C in a 0.1% Collagenase II (97%+, ThermoFisher Scientific, Karlsruhe, Germany) in Gibco DMEM/F12 (1:1) (1X) + GlutaMAX^TM^-I (Life Technologies, Darmstadt, Germany) with 10% FCS (Fisher Scientific GmbH, Schwerte, Germany) and 0.05 mg/mL Gentamicin (10 mg/mL, Capricorn Scientific, Ebsdorfergrund, Germany). Next, cells were filtered through a 100 µm cell strainer, washed, counted with a Neubauer counting chamber, and plated at a cell density of 3000 cells/cm^2^ in standard cell culture flasks in DMEM/F12 (1:1) (1x) + GlutaMAX^TM^-I (Life Technologies, Darmstadt, Germany) supplemented with 10% FCS and 0.05 mg/mL Gentamicin (from here onwards, standard cell culture medium). After the cell count was performed, medium was added until the corresponding cell density of 3000 cells/cm^2^ was reached and then transferred to a cell culture flask. This was also carried out for all cell isolation methods listed below. All isolated cells were subcultured until passage 4 (P4) or used immediately after isolation or subculturing, depending on the experiments.

*Perichondrocytes (explant outgrowth culture (PC-O) and enzymatic digestion (PC-D))*: PC-O were isolated using the primary explant technique [45]. The donor tissue was washed extensively in FCS-free DMEM/F12 medium and fat, and excess connective tissues were removed. Perichondrium was separated from the cartilage and minced into small pieces (<1 mm^3^). Approximately two-thirds of the perichondrium was used for the explant culture. The explants were allowed to adhere to the plastic for a few minutes before the addition of cell culture medium. The medium change was performed every 2–7 days only after observing the first cell outgrowth. Care was taken to prevent the explants from detaching during handling. The cells were grown to 90% confluence, after which the explants and the cells were trypsinized and filtered through a 100 µm cell strainer. The isolated cells (PC-O) were then washed and plated (as passage 0) at cell density 10^4^ cells/cm^2^ in standard cell culture medium.

The remaining one-third of the perichondrium was digested in Liberase DH (Roche, Mannheim, Germany) 1:100 in DPBS with Mg^2+^ and Ca^2+^ (ThermoFisher Scientific, Karlsruhe, Germany) for 30 min at 37 °C. The digested tissue was then passed through a 100 µm cell sieve, and the isolated cells (PC-D) were washed with the medium before being cultured in standard cell culture medium (P0).

For microtia patients, the removal of perichondrium was more difficult due to the extensive tissue around small cartilage islands. Cartilage islands were surrounded by perichondrium as well as connective tissue.

All isolated cells were used until passage 4 (P4) or immediately after isolation, depending on the experiments. Medium change was performed 3 times a week.

### 2.2. Colony Formation Assay

To examine the ability of colony formation in healthy and microtia perichondrocytes, PCs obtained via different isolation methods from healthy and microtia tissues and CC-M (reference control) were plated at a density of 52 cells/cm^2^ (*n* = 3) in a 6-well plate and cultured for 7 days at 37 °C, 5% CO_2_, with regular medium changes performed every 2–3 days. Formed colonies were then fixed with 4% PFA for 30 min before staining with crystal violet (Sigma Aldrich, Darmstadt, Germany, T123.1) overnight at room temperature (RT). Afterwards, the samples were washed with water until the colonies were visible. The number of colonies was counted using a stereomicroscope independently by three certified laboratory technicians to avoid subjective bias, whereby a region of >32 cells was defined as a colony [46].

### 2.3. Migration Assay

The migratory capacity was investigated using the CytoSelect^TM^ 24-Well Cell Migration Assay (Cell Biolabs, Inc., Hölzel Diagnostika, Köln, Germany) according to the manufacturer’s instructions. Briefly, 3·10^5^ cells were resuspended in 300 µL FCS-free standard cell culture medium and seeded in the upper chamber (8 µm pore size) of a 24-well plate. A total of 500 µL of standard cell culture was added to the lower well of the migration plate. After overnight culture, non-migrated cells were removed by using a cotton swab, and migrated cells were stained with the provided solution from the manufacturer. Dye from stained cells were extracted with the provided solution by the manufacturer, and the absorbance was measured at 560 nm with a multimode plate reader (Infinite 200 Pro Plate reader, Tecan Austria GmbH, Grödig, Austria) to determine the number of migrated cells.

### 2.4. Flow Cytometry

The expression of progenitor cell surface markers in healthy auricular PC-O, microtia PC-O, and CCs was analyzed via flow cytometry (BD FACSCanto II, Becton Dickinson, Heidelberg, Germany). The mesenchymal stem cell (MSC) -markers CD90, CD73, CD44, CD105, CD146, and CD166, as well as the integrins α5 (CD49e) and β1 (CD29), were analyzed. All were purchased at Biolegend (BioLegend, Inc., San Diego, CA, USA). Data were analyzed using FlowJo^TM^ software v10.8.1, and for data acquisition, Diva Software v8.0.1 was used. Unstained cell populations were used as gating controls, and isotype controls were used to evaluate background staining. The list of antibodies is shown in Supplemental Table S3.

### 2.5. Quantitave Reverse Transcription Polymerase Chain Reaction (RT-qPCR)

For RT-qPCR, mRNA of cultured cells was isolated and purified using Bioline Kit (Bioline, Meridian Bioscience, Luckenwalde, Germany), according to the manufacturer’s instructions, and stored at −80 °C for later use. The quality and concentration of mRNA were determined by measuring the absorbance at 260 nm and 280 nm on TECAN NanoQuant Plate^TM^ (Tecan Austria GmbH, Austria) using a spectrophotometer (Infinite 200 PRO, Tecan Austria GmbH) and calculating the absorbance ratio. The mRNA samples were treated with DNase (Promega, Walldorf, Germany) for 30 min at 37 °C to eliminate residual DNA contamination, and cDNA was synthesized from 1 µg of total mRNA in a volume of 40 µL using SensiFAST^TM^ cDNA synthesis Kit (Bioline, Meridian Bioscience, Luckenwalde, Germany) according to the manufacturer’s instructions. qPCR was performed using FastStart Essential DNA Probes Master Mix, primers (listed in the Supplemental Table S2), and the corresponding probes from Universal Probes Library on a Roche LightCycler^®^ 96 Instrument (all Roche Diagnostics GmbH, Mannheim, Germany). The amplification protocol was 10 min pre-incubation at 95 °C followed by 45 cycles of 10 s at 95 °C and 30 s at 60 °C. The expression levels of all genes were calculated using the 2^−(ΔCq)^ method relative to the average of 2 reference genes—β-Actin and β2-Microglobulin.

### 2.6. Three-Dimensional Culture

#### 2.6.1. Spheroids

Cells were expanded till passage 2 and afterwards suspended in DMEM/F12 (1:1) (1X) + GlutaMAX^TM^-I (supplemented with 10% FCS and 0.05 mg/mL Gentamicin, 50 ng/mL TGF-β3 (>95%, Proteintech, Planegg-Martinsried, Germany), 50 µg/mL ascorbic acid (>99%, Merck KGaA, Darmstadt, Germany), and 50 µg/mL L-proline (99%, Sigma Aldrich, Darmstadt, Germany)), and 15,000 cells were seeded into each well of an ultra-low attachment plate (Nunclon^TM^ Sphera^TM^ 96-Well, Nunclon Sphera-Treated, U-shaped-Bottom Microplate, ThermoFisher, Karlsruhe, Germany). The medium was filled up to 200 µL and incubated at 37 °C and 5% CO_2_ for 42 days. Medium changes were performed twice a week. At the end of the culture period, 36 spheroids were collected for RNA isolation and qPCR analysis, and 12 spheroids were fixed in 4% formalin and embedded in paraffin for histological and immunohistochemical staining.

#### 2.6.2. Hyaluronan Transglutaminase Alginate (HATG-Alg) Constructs

Cells were expanded till passage 2 to reach a final concentration of 2·10^7^ cells per 1 mL of Bioink. CC-M (*n* = 3), PC-OM (*n* = 3) and healthy PC-OH (*n* = 3) were embedded and cultured in a hyaluronan transglutaminse (HATG)-alginate (Alg) hydrogel (HATG-Alg, 0.5% HATG, 0.25% Alg, 1.5% HA, 2.0% sNAG). sNAG-HATG-Alg-K and sNAG-HATG-Alg-Q were mixed well, and 20 µL of human plasma was added and incubated for 5 min at room temperature to achieve a homogenous crosslinking.

The cells of each donor were washed twice with Tris buffered glucose (TBG) (50 mM TRIS (Sigma Aldrich, Darmstadt, Germany), 200 mM D-Glucose (Sigma Aldrich, Darmstadt, Germany), pH 7.6) and taken up in 20 µL TBG. The cell suspension was added to the bioink and mixed very gently by rotating the suspension. Then, 12.5 µL was added to each sterile PDMS ring (4 mm diameter, 1 mm height, *n* = 3 per donor and celltype, manufactured from cooperation partner, Zürich, Switzerland), and 2 mL of 100 mM calcium chloride (Sigma Aldrich, Darmstadt, Germany) was added to initiate the crosslinking process [47]. After 1 h at 37 °C, the calcium chloride was replaced by DMEM/F12 (1:1) (1X) + GlutaMAX^TM^-I supplemented with 10% FCS and 0.05 mg/mL Gentamicin, 50 ng/mL TGF-β3, 50 µg/mL ascorbic acid and 50 µg/mL L-proline. After 1 day, the PDMS ring was detached from the bottom of the 12-well plate using a spatula. The constructs were kept in culture for 42 days. Medium change was performed twice a week. After 42 days, two of the constructs were embedded in paraffin, and one was cryopreserved prior to histological and immunohistochemical stainings.

### 2.7. Histological and Immunohistochemical Analysis

For immunohistochemistry, paraffin sections (5 µm) were subjected to antigen retrieval with citrate buffer pH 6.0 at 80 °C for 20 min. The sections were then incubated with Proteinase K (Dako, Agilent Technologies, Waldbronn, Germany) and subsequently treated with endogenous peroxidase blocking solution (Dako, Agilent Technologies, Waldbronn, Germany) for 30 min. After blocking with 10% normal sheep serum for 30 min, sections were incubated with a primary antibody against Aggrecan, Elastin, Collagen type 1 or collagen type 2 at 4 °C over night. Used antibodies are shown in Supplemental Table S2. Next, slides were washed in PBS 0.1% Tween 20, and the secondary antibody (biotinylated anti-rabbit or anti-mouse IgG) was added for 45 min. Samples were washed prior to the application of streptavidin-biotinylated horseradish peroxidase complex (GE Healthcare GmbH, Munich, Germany) and visualized with 3-Amino-9-ethylcarbazole (AED) peroxidase substrate solution (Scytek Laboratories, West Logan, UT, USA).

Alcian blue (Carl Roth GmbH + Co. KG, Karlsruhe, Germany) staining was undertaken to visualize sulfated GAGs by immersing the paraffin sections into 1% Alcian blue solution in 3% acetic acid (Merck KGaA, Darmstadt, Germany) (pH 2.5) for 30 min at RT. The sections were then transferred to 3% acetic acid for 1 min and subsequently washed in distilled H_2_O for 2 min before being counterstained with 0.1% nuclear fast red (Carl Roth GmbH + Co. KG, Karlsruhe, Germany). For staining of acidic proteoglycans, 0.1% Safranin-O solution in distilled water was used. First, the slides were stained in Wiegert’s iron hematoxylin working solution (Roth, Karlsruhe, Germany), washed, and then transferred to a fast green solution. Afterwards, the slides were rinsed quickly in acetic acid solution and stained in 0.1% Safranin-O (Roth, Karlsruhe, Germany) solution. For all histological stainings, slides were dehydrated to xylol and mounted with aqueous mounting medium (ScyTek, West Logan, UT, USA).

All tissue sections were imaged using Zeiss Axio Observer Z1 with AxioCam 503 color and analyzed with Zen-software version 2.3 (device and software provided by Carl Zeiss Microscopy GmbH, Oberkochen, Germany).

### 2.8. Statistical Analysis

The optical densities, colonies, mRNA expression levels, and mean fluorescence intensities between the different cell types and microtia cells and healthy cells were compared using two-way ANOVA in GraphPad Prism 9 software. A *p*-value < 0.05 was considered statistically significant.

## 3. Results

### 3.1. Migration and Colony-Forming Capacity of Perichondrocytes from Microtia Patients

**Figure 1:**
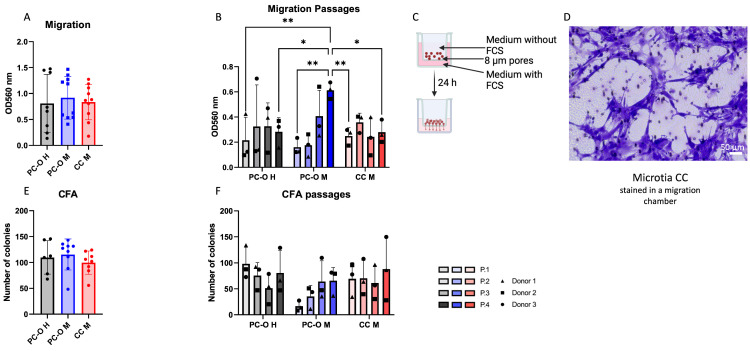
Migration and colony-forming capacity via CFA (colony forming assay). (**A**,**B**) comparison of migration capacity of healthy PC-O (PC-O H), microtia PC-O (PC-O M), and microtia CC (CC M) and the change over four passages ((**B**) where * *p* < 0.05, ** *p* < 0.005). (**C**) Overview of used migration method, created with BioRender.com. (**D**) Stained CC M in migration chamber. (**E**,**F**) Comparison of number of colonies of PC-O H, PC-O M, and CC M and the change over four passages.

High migratory and colony-forming capacity are the two hallmark characteristics of cartilage stem/progenitor cells (CSPCs) [45,48], and they are exhibited by perichondrocytes. Therefore, we analyzed the migration and colony-forming ability of microtia perichondrocytes and compared them with healthy perichondrocytes and microtia chondrocytes. PC-O M (PC outgrowth, microtia) showed similar migratory activity to PC-O H and CC-M (Figure 1A). However, a distinct migratory behavior was observed in PC-O M across cell passages (P1–P4) as compared to the other cell types. The migration of healthy perichondrocytes and microtia chondrocytes remained stable and comparable over four passages. In contrast, PC-M displayed lower migratory activity in P1 compared to the other cell types, followed by a gradual increase in migration with each passage (Figure 1B). By P4, the migration of PC-O M was significantly higher than that of PC-O H and CC M in the same passage.

The ability to form colonies was similarly pronounced in all three cell types (Figure 1E). When considering cell passages, PC-O M showed lower colony formation in P1 than the other cell types but increased to a level comparable with PC-O H and CC-M at P4.

### 3.2. Influence of the Isolation Method on Migration and Colony-Forming Capacity of Microtia Perichondrocytes

**Figure 2:**
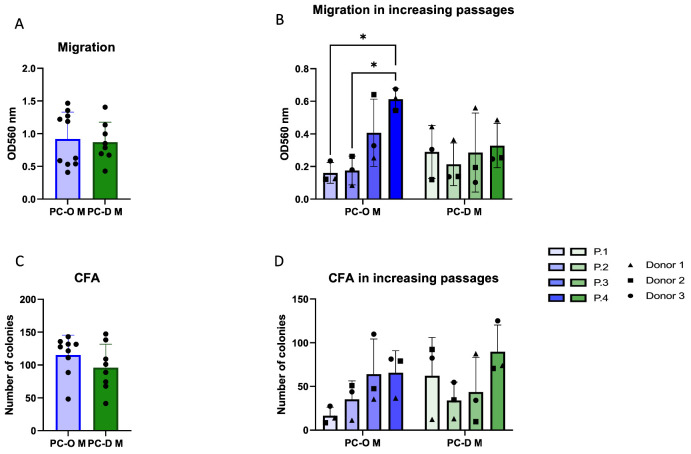
Comparison of microtia perichondrocytes obtained via two different isolation methods. PC-O were obtained via explant culture, while PC-D were obtained via digestion of microtia perichondrium. Overall migration (**A**); change in migration ability over 4 passages with * *p* < 0.05 (**B**); colony-forming capacity overall (**C**); and change over four passages (**D**).

Explant outgrowth culture is an established means of CSPC isolation from cartilage. To determine if the outgrowth from microtia perichondrium would similarly result in an enriched population of progenitor-like cells, we compared microtia perichondrocytes isolated via enzymatic digestion (PC-D M) and explant outgrowth culture (PC-O M) with regard to their migration and colony-forming ability. On average, no significant difference in migration or colony-forming ability (Figure 2A,C) was found between PC-M obtained via these two isolation methods. However, when passages were considered, PC-D M showed stable migratory activity across passages, whereas PC-O M displayed low migration in the first passage, followed by a gradual increase until P4, where it exceeded the migration of PC-D M (Figure 2B). An overall tendency for an increase in the colony-forming capacity was observed in both PC-O M and PC-D M; however, in P1, the cells, isolated by enzymatic digestion, formed more than twice as many colonies as PC-O M (Figure 2D).

### 3.3. Cartilage-Related Gene Expression in 2D Culture

**Figure 3:**
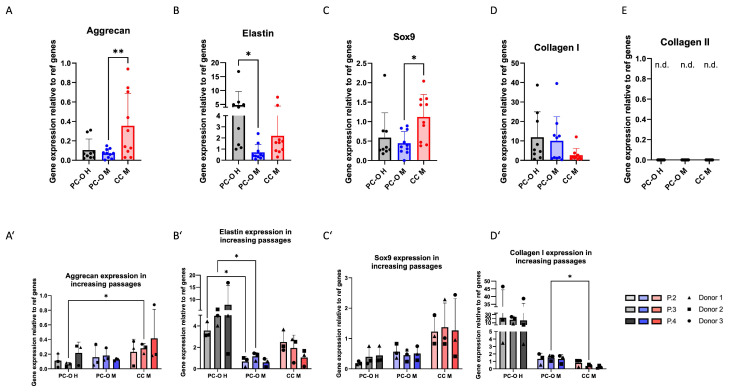
Comparison of mRNA expression of cartilage-related genes of PC-O H, PC-O M, and CC M. Aggrecan (**A**); aggrecan over three passages (**A’**); elastin (**B**) over three passages (**B’**); sox 9 (**C**); sox9 over three passages (**C’**); collagen I (**D**); collagen I over three passages (**D’**); collagen II (**E**); n.d.—not detected. The expression levels of all genes were calculated using the 2^−(ΔCq)^ method relative to the average of two reference genes: β-actin and β2-microglobulin ((**A**–**D**) *n* = 10; (**A’**–**D’**) *n* = 3; * *p* < 0.05 and ** *p* < 0.005; n.d. = not detectable).

Gene expression of cartilage-related genes elastin, aggrecan, sox9, collagen I, and collagen II was investigated in all three cell types. In each case, the gene expression of the PC-O of the healthy donors was compared with the PC-O and CC from microtia samples. The expression of aggrecan and sox9 were significantly higher in CC-M compared to perichondrocytes but similar in microtia and healthy perichondrocytes. A significantly higher expression of elastin in healthy PC-O than in PC-OM and microtia CC was seen. Collagen I was similarly expressed in microtia and healthy perichondrocytes, but, in both cell types, it was higher than in microtia chondrocytes. Collagen II expression was not detected in any of the investigated cell types (Figure 3A–D).

The expression of all before-mentioned genes was compared in passages two, three, and four. Since the passaging of the cells prior to 3D culture is a necessary step for reaching enough cells for tissue engineering approaches, we focused on differences in higher passages, beginning from passage 2. Elastin expression in PC-O H increased over the passages, whereas it remained at very low levels in PC-O M and decreased in CC M (Figure 3B). In contrast, aggrecan expression increased in CC M with higher passage but remained relatively constant in both healthy and microtia PC-O (Figure 3A’). Gene expression levels of *sox9* were unaltered over four passages in all cell types, remaining highest in CC M. Collagen I expression was highest in PC-O of healthy donors, decreased in CC M, and remained constant in PC-O M. The collagen I mRNA level of PC-O M in P3 was significantly higher than in CC M of the same passage (Figure 3A’–D’).

**Figure 4:**
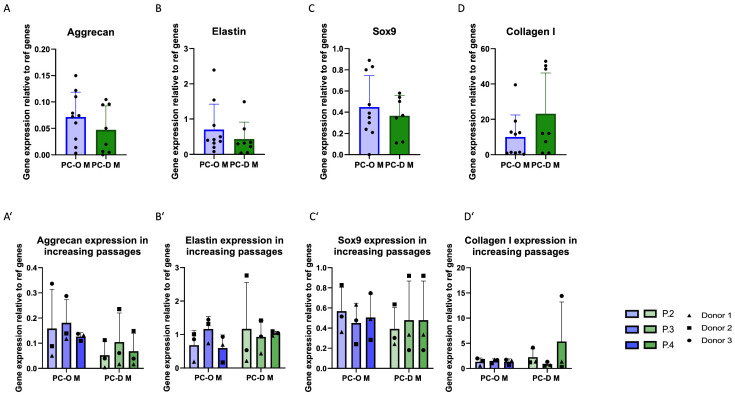
Comparison of mRNA expression of cartilage-related genes of PC-O M and PC-D M. Aggrecan (**A**); aggrecan over three passages (**A’**); elastin (**B**) over three passages (**B’**); sox 9 (**C**); sox9 over three passages (**C’**); collagen I (**D**); collagen I over three passages (**D’**). The expression levels of all genes were calculated using the 2^−(ΔCq)^ method relative to the average of two reference genes: β-actin and β2-microglobulin.

When comparing PCs obtained via outgrowth with enzymatic digestion, minor differences were found in mRNA levels of elastin, aggrecan, and *Sox9*, with slightly lower values in the PC-D (Figure 4A–C). In contrast, collagen I expression was almost twofold higher (albeit statistically non-significant in PC-D M as compared to PC-O M (Figure 4D)). Relatively small and non-significant changes in the expression of all genes were found across passages in both PC-D and PC-O (Figure 4A–D).

### 3.4. Surface Marker Profile of Different Cell Types Is Similar in Flow Cytometry

**Figure 5:**
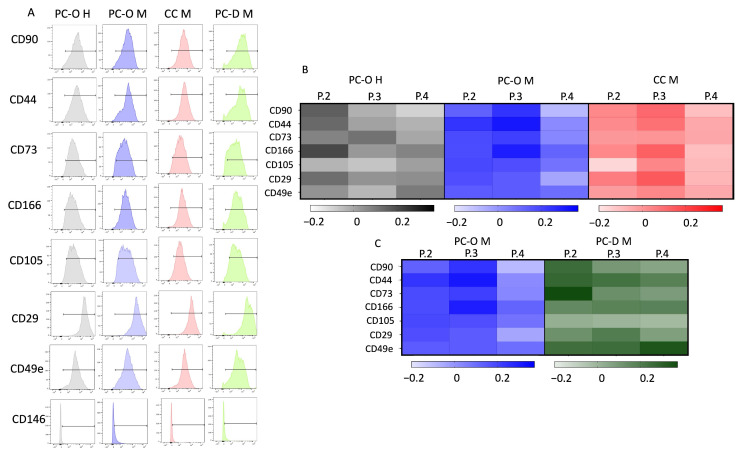
Flow cytometry results. (**A**) shows detection of respective surface markers. Detection of all mesenchymal stem cells markers with over 90% except for CD146. Normalized mean fluorescence intensity (MFI) to P1 with lg of respective MFI with heat map. Darker color is equal to a higher expression in comparison to P1. Comparison of PC-O H, PC-O M, and CC M (**B**); comparison of MFI of PC-O M and PC-D M (**C**).

The most common surface markers of mesenchymal stem cells that are, meanwhile, also used to characterize chondrogenic progenitor cells were analyzed for their presence on the cells via flow cytometry. Markers were selected from the review of Jessop et al. [44]. All analyzed markers and integrins were expressed on all three analyzed cell types, except CD146, which was not found on either of the PCs or CC-M (Figure 5A). Both healthy and microtia PCs, as well as CC-M, contained over 90% of cells positive for CD90, CD44, CD73, CD166, CD105, and the integrins CD49e and CD29, with only little variation among the cell types.

Since the percentage of cells did not change during the cell culture passages, we decided to investigate the intensity of surface marker expression after cell passaging using the mean fluorescence intensity (MFI) of MSC surface markers in all cell types across four passages. Several trends emerged from this analysis. In healthy PC-O, CD90 was highest at P2 but decreased with further passages, whereas a slight increase in the MFI of CD90 was observed in PC-O M and CC M until P3, followed by a decrease in P4 (Figure 5B). Similarly, the MFI of CD44 was at its highest in P2 of PC-O H but increased until P3 in PC-O M and CC-M. CD73 expression was generally higher in healthy vs. microtia PC-O, although a dependence of passages could not be detected in all cell types. The integrins CD49e and CD29 and the MSC markers CD105 and CD166 were largely consistently expressed across the four passages in all cell types. Looking at the logarithms of the heat map normalized to passage 1, certain trends emerge (Figure 4). There was a decrease in integrins and CD90 in microtia perichondrocytes.

Comparison of PC-O with PC-D revealed no significant differences in the expression of progenitor surface markers. The MFI levels in general barely differ; even the trends were very similar. CD90, CD44, CD73, and CD49e did not yet appear to be as highly expressed in PC-D M at passage 1, again with strong donor dependence. From passage 2 onwards, the investigated markers remained largely constant, which means that a passage-dependent change of the surface molecules could not be detected.

Flow cytometric analysis of the enzymatically obtained cells from the cartilage tissue of microtia patients immediately after isolation revealed that the stem cell markers are not yet strongly expressed. CD90 (range: 0.5–1%) could not be detected at all, and CD44 (range: 25–37%) and CD29 (range: 62–78%) were at a low level. Due to the low number of cells directly after isolation, we focused on the most important markers.

### 3.5. Matrix Production within 3D Spheroids

**Figure 6:**
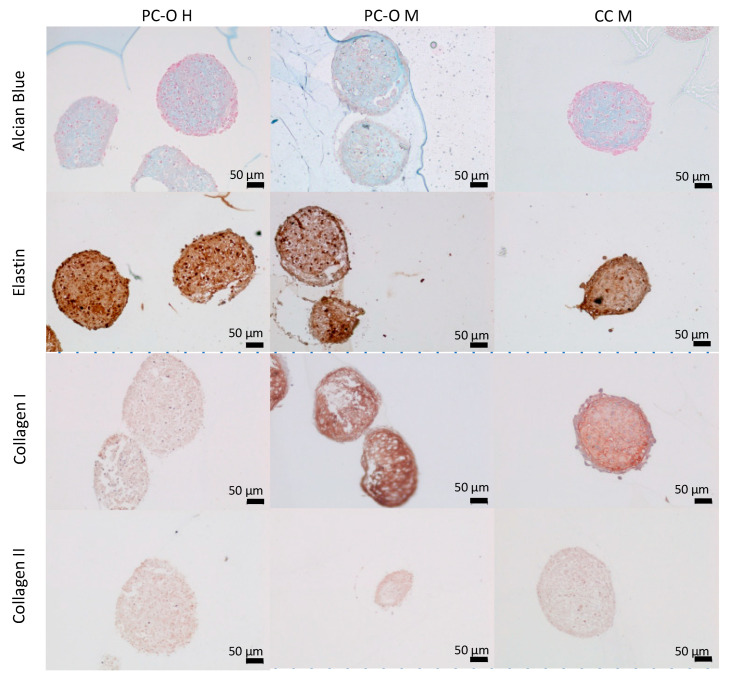
Immunohistochemical staining of spheroids. Cells were cultured in a 96-well ultra-low-attachment plate in chondrogenic medium for 42 days. Staining with Alcian blue for glycosaminoglycans (first row) and antibodies for elastic cartilage specific compounds (elastin, second row; collagen I, third row; and collagen II, fourth row).

To investigate the properties of all three cell types in a three-dimensional environment, they were cultured as spheroids for 5 weeks. All three cell types formed compact, round-shaped spheroids. Elastin, collagen I, and collagen II expression was detected in all spheroid cultures, but more intense staining was observed in spheroids formed by CC M as compared to PC. We observed an expression of glycosaminoglycans through Alcian blue staining. The formation of lacunary structures containing isogenic groups of cells was observed in all spheroids. In contrast to the previous reports, we did not observe a disorganized structure in spheroids from microtia patients, including those formed by CC M (Figure 6).

### 3.6. Formation of Chondrogenic Matrix in HATG-Alg Hydrogels

**Figure 7:**
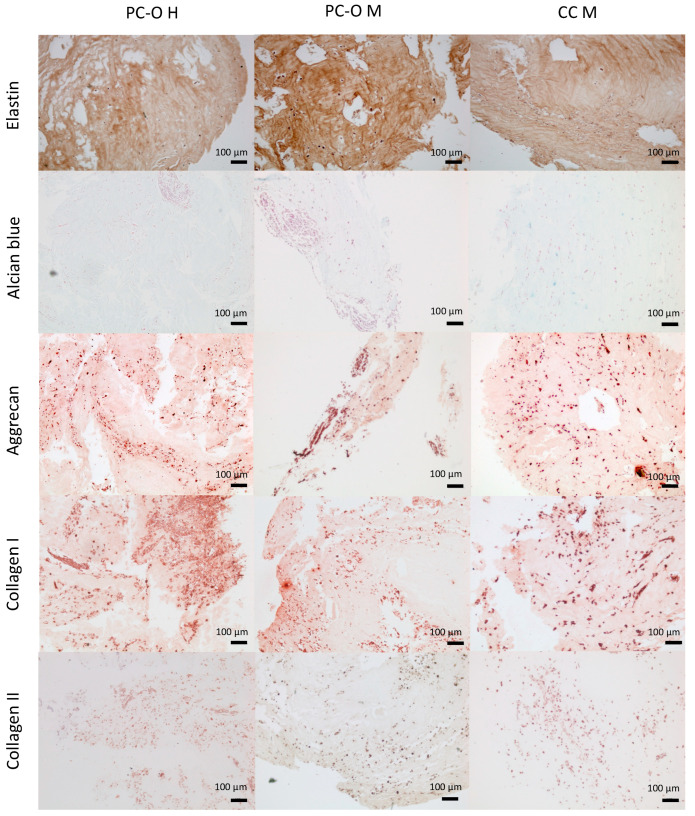
Immunohistochemical staining of HATG-Alg-constructs. Cells were embedded into a HATG-Alg Bioink and cultured for 42 days in chondrogenic medium. Staining with Alcian blue for glycosaminoglycans (second row) and antibodies for elastic cartilage specific compounds (elastin, first row; aggrecan, third row; collagen I, fourth row; and collagen II, fifth row).

As these cells are to be used in a bioprinting procedure with the HATG-Alg hydrogel, we cultured the three cell types in a scaffold-based 3D model using HATG-Alg hydrogel as a scaffold to observe whether the cells start to express cartilage-specific proteins. The constructs were cultured for 5 weeks. Elastin was highly positive in all three cell types. Aggrecan was also detected via immunohistochemical staining. Especially where cells are gathering, the staining for aggrecan was positive. Also, collagen I expression was detected in all discs after 5 weeks of in vitro culture. Increased formation of collagen I occurred mainly where more cells accumulated. This is most impressive in Figure 6. Collagen II production was also detected via immunohistochemistry. But also, for microtia CC, production of collagen II could be detected (Figure 7). Healthy perichondrocytes also produce collagen II when enough cells are near each other (Figure 7).

## 4. Discussion

Several studies have been conducted on the reconstruction of ear cartilage using tissue engineering techniques. In most of these studies, chondrocytes isolated from different regions of the human body, such as the nose, ribs, and ears, were used [49,50,51,52,53,54]. It is well known that CCs dedifferentiate in a monolayer (2D) cell culture [55]. Studies have been performed to investigate the phenotypic and cytoskeletal changes [30]. The study by Zhou et al. in 2018 counts as a pioneering study using microtia CC as a cell source for an autologous tissue engineering approach [26]. Zhou et al. first used isolated and in-cell-culture-expanded microtic chondrocytes for the autologous reconstruction of the human auricle in children. It stands to reason that PCs also dedifferentiate in 2D culture. Togo et al. showed redifferentiation of rabbit PC in higher passages, but has not yet been fully investigated [55]. It is necessary to investigate the optimal combination of cytokines for redifferentiation [55]. PCs show a more fibroblast-like type from the beginning. However, none of the previously mentioned studies focused on the regenerative potential of the perichondrium. Kobayashi et al. postulated the presence of progenitor cells in the human perichondrium for the first time [40]. The first use of PCs in an animal model was performed by Kagimoto et al. in 2016 [38]; they showed the possible regenerative potential of perichondrocytes in a xenograft setting.

In this study, the progenitor-like properties and behavior of isolated cells from the perichondrium of microtia patients and healthy donors were compared in a monolayer culture until passage 4, which is the number of passages the redifferentiation potential of the cells is retained as, as well in 3D culture systems. We isolated cells from the perichondrium as well as from cartilage from microtia patients and the perichondrium of healthy donors. We were able to culture them for at least four passages without a decrease in growth rate. Progenitor-like properties include proliferative capacity and the ability to differentiate into different cell types within a tissue [56]. Progenitor-like properties were evaluated using a colony-formation assay (CFA), a migration assay, and a surface marker analysis for mesenchymal stem cell markers which are also expressed on chondrogenic progenitor cells. The CytoSelect24TM^®^ Assay was used for migration experiments due to the high reproducibility of the results. For our approach to investigating migration in this study, this assay is precise and reproducible enough, although there might be more precise methods with which to analyze migration in future studies [57]. The three cell types were isolated from three donors each (microtia patients and healthy donors) and cultivated in cell culture over four passages to investigate whether the properties of the progenitor cells were retained during the expansion period. We showed that these properties were at least maintained over four passages in all three cell types; the microtic perichondrocytes even showed an increasing migratory capacity during the four passages. Migrative properties are, in the case of tissue engineering, a selection criterion with which to yield progenitor cells [45]. In our study, migrative properties are present in our target cells PC-O M, which indicates an important property of these cell types for TE applications. This is an important component for the suitability of PC-O M for the reconstruction of auricles using TE methods. In our study, CC M and PC-O M were not showing a significant difference with regard to the number of colonies, but there is a slightly lower number in case of CC M compared to PC-O M, whereas other studies showed different colony-forming abilities of CC and PC [40]; they showed that PC forms significantly more colonies than CC [40]. In our comparison of colonies formed during different passages, it was shown that PC-O M formed significantly fewer colonies in the first passage than CC M or PC-O H, but the number of colony-forming cells of PC-O M increased during the expansion time. In the course of this study, all cell types analyzed seem to converge to one level through investigation of the colony forming assay. This is a further hint that PC-O M has similar progenitor-like cell feature compared to PC-O H and CC M. By interpreting the results of the colony forming assay, it seems as though the cells can be used for up to four passages for autologous reconstruction/TE approaches.

We demonstrated that PC and CC express selected MSC specific markers CD90, CD44, CD73, CD166 and CD105 on their surface. CD146 was not detected on the analyzed cells, which, according to the present literature, has, until now, only been observed on chondrogenic progenitor cells isolated from articular cartilage [44]. Also, Integrins α5 and β1 could be detected at high levels, similar to other studies such as Koboyashi et al., Otto et al. and Guasti et al. [33,40,56]. Flow cytometric analysis of surface molecules demonstrated decreasing expression of integrins by normalizing to passage 1 for PC-O M. It is already known that there is a negative correlation between the expression of integrins and the ability of migration [58]. Wang and Thampatty have shown that integrin β1 plays a role in the migration ability of cells. An antiproportional relationship between migratory capacity and integrin β1 expression is expected [59]. Lower levels of integrin β1 result in a higher migration ability. This is in common in migration and flow cytometric analysis data. We showed that PC-O M exhibits a decrease in integrin β1 expression with increasing passage, with concomitant increased migratory ability from passage 3. This might be a sign of an ongoing dedifferentiation but does not affect the planned purpose for auricular reconstruction. Integrin a5 seems to stay on one level, which is a sign of an incomplete colocalization of the two markers. The number of positive cells does not decrease; only the number of antigens on each cell shows a decrease via corresponding signal intensity. The biological importance must be evaluated in further experiments [26,43].

Gene expression analysis showed that CCs derived from microtia patients show a more chondrogenic expression profile than PCs from healthy donors and microtia patients. Detail analysis showed that chondrocytes express more aggrecan and also sox9, whereas elastin is more expressed in the healthy perichondrium. Sox9 is considered to be an important marker for chondrogenic progenitors by promoting chondrogenic commitment [60]. These findings are in line with the observations of other groups [36,37,40,41,61,62,63,64]. 

This is also consistent with the immunohistochemical staining for the corresponding proteins found in paraffin sections of microtic cartilage. In the perichondrium, increased elastic fibers are detectable, while in the cartilage itself, aggrecan is highly expressed. PC-O from microtia patients showed a significantly decreased gene expression of elastin compared to PC-O H. We assume that the dedifferentiation process which is undertaken in monolayer cultures leads to lower expression of cartilage-specific genes, which is in line with the existing literature [14,41,42,61].

In healthy cartilage, a high proportion of collagen II can be observed in native paraffin sections, but this could not be detected in the gene expression analysis of monolayer cultured cells, which is in line with the known literature. Ciorba et al. showed a switch from collagen II expression to collagen I expression because of the dedifferentiation process during cell culture [62]. Collagen I is found in higher amounts around the lacunae in the cartilaginous part and in the perichondrium of healthy donors and microtia patients. A dependence of passages on the gene expression could not be demonstrated. Gene expression levels of aggrecan, collagen I, and elastin compared to housekeeping genes remain constant until passage 4. In some studies, a collagen II expression in lower passages was shown [55], as well as decreasing levels of collagen II with higher passages [61]. A significant difference in gene expression of elastin from PC-O H and PC-O M could be shown when comparing the passages. One reason could be the high amount of fibrous tissue compared to healthy cartilage, which is connected to the microtia perichondrium; therefore, more fibroblast-like cells were isolated during cell isolation. In contrast to other studies, we could not show an increase in collagen I expression during culture, which seems to be a main characteristic of the dedifferentiation process in vitro [61,62]. It seems that immediately after isolation and cultivation in 2D cell culture, our cells start to express collagen I at a very high level, which could not be increased during the rest of the cell culture period.

We assessed two different 3D cell culture systems (spheroid model and scaffold/matrix-based model). Because the cells in native cartilage are sporadically embedded in the ECM, we tested a hydrogel model as cells should be used in an autologous reconstruction of the auricle by, e.g., bioprinting. The used hydrogel was developed by a cooperation partner in the SNF project (Sinergia-Project: 2-77120-17) and should therefore be tested for suitability for cartilage reconstruction with human chondrocytes and perichondrocytes. The used hydrogel also contains alginate. Alginate is often used to fabricate a cross-linked hydrogel in tissue engineering approaches, mainly for embedding cells into the matrix [65]. The hydrogel used has the advantage—for this aim of the study—to contain components of native cartilage (hyaluronic acid). Like in the native elastic cartilage, the cells are also separated from each other in the hyaluronic acid matrix. Since hyaluronic acid, as a GAG, is a component of native cartilage [66], it stands to reason that cells will start to produce cartilage-specific matrix again. This is shown in Figure 6. Aggrecan, as a prominent cartilage-specific protein which is covalently bound to sGAGs, is expressed in the HATG Bioink model. This is especially seen when cells are gathering but not in cell-free areas or where only a low cell density can be found. Since histological stainings are not as sensitive as immunohistochemical stainings, the weak Alcian blue staining confirms the findings of this study. It stands to reason that a longer incubation period results in a higher expression and distribution of sGAGs, which would result in a stronger staining. Due to the higher cell density in spheroids compared to the hydrogel model, the response in the 3D model may be different. For the bioprinting approach, the hydrogel model is generally preferred, but it requires many more cells than a spheroid model. The spheroid model is the model of choice as a pretest for the general suitability of donor cells for bioprinting. If the cells were also gathering, PC-O H, and PC-O M expressed collagen II, the reason for the gathering of cells remains unclear so far. Also, in a recent publication, collagen II was found but only close to the location of the cells. Other studies showed, in in vitro experiments, only collagen II expression for chondrocytes [67]. Bioprinting experiments require a very high cell number, which makes it necessary to culture cells for several passages. For microtia perichondrocytes, however, a significantly increased migration capacity has been demonstrated in passage 3 in our study. However, migration could be reduced by crosslinking the bioinks. Nevertheless, it was not possible to obtain a homogeneous cell distribution in the 3D constructs. Thus, there are always areas where cell gathering is increased and collagen II is produced, but also areas where the cells were not close enough to each other to detect collagen II production via immunohistochemistry. Our 3D constructs were kept in culture for 6 weeks. It could be possible with an extended culture period that the cells start to produce more collagen II. This would be in line with the current literature [42]. Spheroid culture also showed collagen II production in all cell types examined. However, after 6 weeks, collagen I was still much more present, maybe due to protein stability. Another reason for the strong staining for collagen I in the HATG Bioink model and the spheroid model could be that the enzymes that would be required for protein degradation of collagen I are expressed or secreted in the cell culture to an insufficient extent or are completely absent. Collagen II production has also been shown in previous studies, but only after at least 3 weeks in vivo [41]. When comparing healthy and microtia chondrocytes, it could be shown that the constructs seeded with healthy cells had a higher collagen II expression than the constructs seeded with microtia cells [41]. In our study, collagen II expression of microtia perichondrocytes was detected via immunohistochemistry, albeit significantly less than expected. Despite the use of a chondrocyte differentiation medium, collagen II is not produced at a large level. Further studies would be needed to test the extent to which collagen II production can be increased using a different, more potent differentiation medium to obtain a more cartilage-like structure. We also investigated whether the spheroids show structural differences in vitro. In our study, no abnormalities in the structure of spheroids were found after 6 weeks of in vitro culture, while Zucchelli et al. showed that a disorganized structure is formed in spheroids from microtia cartilage stem/progenitor cells [42]. However, this group used significantly more cells per spheroid and a different method of spheroid formation than our group. In a further study, we could investigate whether structural changes could be detected with more cells per spheroid and whether the expression profile would then also change.

## 5. Conclusions

This study demonstrated that perichondrocytes from microtia patients have chondrogenic properties and start to produce cartilage matrix in in vitro 3D models to the same extent as healthy perichondrocytes and chondrocytes from microtic cartilage tissue. Microtic perichondrocytes showed the same migration, colony-forming capacity, gene expression profile, and surface marker profile as healthy perichondrocytes and microtia chondrocytes and maintained these properties up to passage 4. In 3D culture systems, all cells were shown to start producing cartilage-specific proteins after 42 days in culture. In monolayer cultures, there was no significant difference in cell behavior between the different isolation methods. However, isolation was more successful using the explant technique. This is why it is recommended to use explant culture for routine approaches. Based on the results of this study, perichondrocytes from microtia patients are a suitable—at least, as an additional—cell source for TE applications using autologous cells. Since donor dependence was observed and chondrocytes from microtia patients could start to form a disorganized tissue, as shown in the study by Zucchelli et al. [42], the cells should be tested for their usability in in vitro models before they are used for tissue engineering methods, e.g., for printing an ear for transplantation purposes. The next step will be to increase the cell culture time of the 3D models to observe whether cartilage redifferentiation in vitro becomes more complete, such as degradation of collagen I and increased expression and secretion of collagen II and elastin. In addition, we will investigate whether the distribution of cartilage-specific components such as collagen II or proteoglycans in the bioink model can be improved by increasing the cell culture time, and whether the hydrogel degrades over time.

## Data Availability

The data supporting the findings of this study are available within this paper and the Supplementary Material. Additional data are available from the corresponding author upon reasonable request.

## Supplementary Materials

The following supporting information can be downloaded at https://www.mdpi.com/article/10.3390/cells13020141/s1: Table S1: Primers for qPCR; Table S2: Antibodies for immunohistochemistry; Table S3: Antibodies for flow cytometry.
