# Supplementary material for: Suitability of Ex Vivo-Expanded Microtic Perichondrocytes for Auricular Reconstruction
Source: Cells. 2024 Jan 12;13(2):141. doi: 10.3390/cells13020141 (PMC10814984; doi:10.3390/cells13020141)
Supplement: Supplementary file 1 [file cells-13-00141-s001.zip › cells-2764984-supplementary.pdf]

Table S1. Primers for qPCR.

Gene	s or as	Sequence	UPL Probe
**B2M**	s	TTCTGGCCTGGAGGCTAT	12
**B2M**	as	TCAGGAAATTTGACTTTCCATTC	12
**β-Actin**	s	ATTGGCAATGAGCGGTTC	11
**β-Actin**	as	CGTGGATGCCACAGGACT	11
**SOX9**	s	TCTCGCTCTCGTTCAGAAGTC	61
**SOX9**	as	GTACCCGCACTTGCACAAC	61
**ACAN**	s	CCTCCCCTTCACGTGTAAAA	76
**ACAN**	as	GCTCCGCTTCTGTAGTCTGC	76
**Elastin**	s	GGAGGTGTTCCCGGAGTC	27
**Elastin**	as	GGTCCCCACTCCGTACTTG	27
**COL2A1**	s	GTGAACCTGGTGTCTCTGGTC	19
**COL2A1**	as	TTTCCAGGTTTTCCAGCTTC	19
**COL1A1**	s	GGGATTCCCTGGACCTAAAG	67
**COL1A1**	as	GGAACACCTCGCTCTCCA	67

Table S2. Antibodies for immunohistochemistry.

**Antibody**	**Species**	**Dilution**	**Enzyme digestion**	**Isotype**	**Manufacturer**
**Aggrecan, polyclonal**	Rabbit	1:100	Pepsin 2 µg/mL in TRIS-HCl (1 mg/mL), pH 2.0	Rabbit IgG, polyclonal	Merck KGaA, Germany-Darmstadt
**Collagen I, polyclonal, #34710**	Rabbit	1:200	Proteinase K	Rabbit IgG, polyclonal	Abcam, Berlin, Germany
**Collagen II, II-II6B3, Monoclonal**	Mouse	1:100	Citrat + Proteinase K	MIgG1, kappa light chain	DSHB 3822
**Elastin, polyclonal, #23747**	Rabbit	1:400	TRIS-EDTA/Citrat	Rabbit IgG, polyclonal	Abcam, Berlin Germany

Table S3. Antibodies for Flow Cytometry.

**Antibody for Flow Cytometry**	**clone**	**Isotype**	**Manufacturer**
**APC anti-human CD29**	TS2/16	Mouse IgG1,	BioLegend, US-San Diego
**PerCP anti-mouse/human CD44**	IM7	Rat IgG2b,	BioLegend, US-San Diego
**PE anti-human CD49e**	NKI-SAM-1	Mouse IgG2b,	BioLegend, US-San Diego
**eFlour™ 450 anti-human CD73**	AD2	IgG1,	eBioscience, ThermoFischer Scientific, Germany - Schwerte
**FITC anti-human CD90**	5E10	Mouse IgG1,	BioLegend, US-San Diego
**FITC anti-human CD105**	43A3	Mouse IgG1,	BioLegend, US-San Diego
**PE anti-human CD146**	P1H12;	Mouse IgG1,	BioLegend, US-San Diego
**APC/Fire™ 750 anti-human CD166**	3A6	Mouse IgG1,	BioLegend, US-San Diego
